# Effect of Peripheral Edema on Oscillometric Blood Pressure Measurement

**DOI:** 10.15171/jcvtr.2014.015

**Published:** 2014-12-30

**Authors:** Shamsi Ghaffari, Majid Malaki, Afshin Rezaeifar, Shahin Abdollahi Fakhim

**Affiliations:** ^1^Cardiovascular Research Center, Tabriz University of Medical Sciences, Tabriz, Iran; ^2^Pediatric Health Research Center, Tabriz University of Medical Sciences, Tabriz, Iran; ^3^Department of Otorhinolaryngology, Tabriz University of Medical Sciences, Tabriz, Iran

**Keywords:** Oscillometric, Edema, Auscultatory, Blood Pressure

## Abstract

***Introduction:*** Blood pressure (BP) measurement is essential for epidemiological studies and clinical decisions. It seems that tissue characteristics can affect BP results and we try to find edema effect on BP results taken by different methods.

***Methods:*** BP of 55 children before open heart surgery were measured and compared according to three methods: Arterial as standard and reference, oscillometric and auscultatory methods. Peripheral edema as a tissue characteristic was defined in higher than +2 as marked edema and in equal or lower than +2 as no edema. Statistical analyses: data was expressed as Mean and 95% of confidence interval (CI 95%). Comparison of two groups was performed by T independent test and of more than two groups by ANOVA test. Mann–Whitney U and paired T-test were used for serially comparisons of changes. P less than 0.05 was considered significant.

***Results:*** Fifty five children aged 29.4±3.9 months were divided into two groups: 10 children with peripheral edema beyond +2 and 45 cases without edema. Oscillometric method overestimated systolic BP and the Mean (CI 95%) difference of oscillometric to arterial was 4.8 (8/-1, P=0.02) in edematous and 4.2 (7/1, p=0.004) in non edematous. Oscillometric method underestimated diastolic BP as -9 (-1.8/-16.5, P=0.03) in edematous group and 2.6 (-0.7/+5, P= 0.2) in non edematous compared to arterial method.

***Conclusion:*** Oscillometric device standards cannot cover all specific clinical conditions. It underestimates diastolic BP significantly in edematous children, which was 9.2 mmHg in average beyond the acceptable standards.

## Introduction

Accuracy of blood pressure (BP) values as a vital medical information is a debating medical issue. The importance of ever-increasing standards of BP measurements is related to the improvement in growingly sophisticated epidemiological studies and improved classifications of hypertension severity.^1^ Device quality is an essential factor for getting the most appropriate results besides staff skill and attention which faces researcher to cuff/stethoscope method, as multiple-observer and methodological errors such as digit preferences, inattention, too rapid cuff deflation and hearing deficits may occur or selection of a single beat for measurement may involve when there are beat-to-beat variations in the pulses and sequential rather than simultaneous comparisons.^2^

Estimation of BP in oscillometric method is an engineered challenge to detect small pressure changes within cuff for the most reliable results compatible with auscultatory and arterial method.^1^Oscillometric validation standard was defined as differences no greater than ± 5 mmHg.^3,4^ Inaccuracy of oscillometric devices to detect BP in certain conditions as in critically patients has been known previously.^5^ Other factors can also affect the accuracy of BP measurement, as in non invasive methods factors such as tissue changes, which can be seen in arterial wall stiffness, and other changes that may occur in scleroderma, all can influence BP results detected by oscillometric and auscultatory.^6^ Edema is a prototype clinical manifestation due to the accumulation of fluid within the interstitial spaces of the body, a common and important sign among patients. Edema can be observed especially in heart failure patients as considerable cases, who need to monitor their BP in home, office, or hospital.

This study tries to find accuracy of BP, measured by invasive (arterial) and non invasive (oscillometric and auscultatory) methods in edematous condition in children, candidate for elective heart surgery.

## Materials and methods

Ethical aspects of this study were confirmed and committee approval was obtained. The work was based on an analytical cross-sectional study, not imposing financial or body harms to patients. All data were kept confidential and was explained only for those eligible patients who had filled consent form before entering the study. At first stage, documents of the subjects with congenital heart disease were reviewed to get information regarding their heart defects as well as their past medical history.

Their history of drug uptake and heart abnormality besides their anthropometric characteristics and their sex and age were recorded in a questionnaire. Exclusion criteria were: hypotension defined as BP below the fifth percentile or below two standard deviations (SDs) of the mean for age and gender^7^ and decreased perfusion of tissues. Patients with peripheral vascular disease or coarctation aorta and infants below six months of age were also excluded.

Children’s BP was obtained by auscultation with standard cuff (the cuff should be placed in a distance at least 40% above the elbow towards the acromion and the width of cuff should cover 2/3 of arm circumference). The definition of BP is based on five kortokof sounds; the first Kortokof sound was considered as systolic BP and the fifth as diastolic.

For arterial BP measurement, a cannula sized up 22 G to 25 G with regard to patients size and age was inserted in radialis artery connected to tubing containing a continuous column of saline which conducted the pressure wave to the transducer. The arterial line was also connected to a flushing system consisting saline and added heparin. Children’s BP was also measured by oscillometric method with suitable cuff based on age and body bulk. All devices were the products of Datex Ohmeda Company. All non invasive BP techniques (oscillometric and auscultatory) were performed by a skilled nurse in operating room after children sedation. All collected results were matched and recorded by arterial method performed by anesthesiologist. Edema as an essential sign was classified in standard grade by a physician as follows:

+1 = a normal foot and leg contour with a barely perceptible pit; +2 = fairly normal lower extremity contours with a moderately deep pit; +3 = obvious foot and leg swelling with a deep pit; +4 = severe foot and leg swelling that distorts the normal contours with a deep pit.

We considered children moderate to the severe peripheral edema or the counter more than 2+ as presence of generalized edema whether it can change body contour or not.

Statistical analyses: All data were shown as Mean ± standard deviation (SD) and 95% of confidence interval (CI). For comparison of mean of BP that was measured by different methods in edema and non edema groups, T independent test was used, while for serially measurements, paired T test were used. For comparison of BP for more than two groups, ANOVA and post hoc analysis or Tukey test were used. All analyses were performed by SPSS 16.00 and P less than 0.05 was considered significant.

## Results

Fifty five children including 34 male and 21 female with mean±SD (Min, Max) age of 29.4 ±3.9 months (7, 144) were entered to the study. Ten out of 55 had peripheral edema more than +2.

Edematous condition as an independent factor does not affect BP values in both systolic and diastolic components. In oscillometric methods, diastolic BP detected in edematous patients was lower than systolic BP, but this difference (mean, 95% CI), 8 mmHg (-0.5/+16, P=0.06) was not significant (Table 1).

**Table 1:** Comparison of blood pressure in three methods in edematous and non edematous children

	**Device type**	**Edema**	**P, Difference (CI95%) Total**	**Non edema**	**Total**
Systole	Arterial	84±18	0.6, D=2.5 (-8/+13) mmHg	86±15	86±15
	Oscillometric	89±15	0.7, D=2.1 (-8/+13) mmHg	91±15	91±15
	Auscultatory	85±15	0.4, D=4.1 (-7/+15) mmHg	89±15	89±15
Diastole	Arterial	56±8	0.6, D=1.4 (-5/+8) mmHg	57±10	57±10
	Oscillometric	47±4	0.06, D=8 (-0.5/+16) mmHg	55±12	55±12
	Auscultatory	54±11	0.3, D=4.7 (-5/+14) mmHg	59±13	59±13

**Figure 1:**
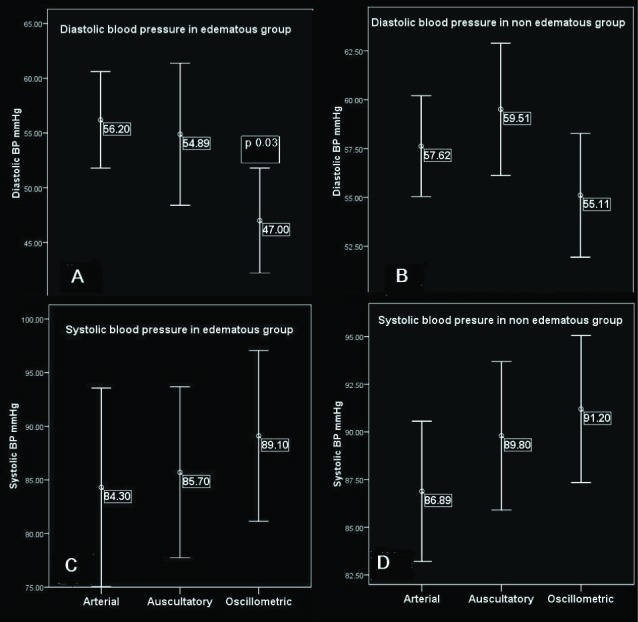
(A-D) comparison of blood pressure, Mean± standard deviation in three methods for systolic and diastolic component in both edematous (A-C) and non edematous (B-D) groups

This comparison was also done for different methods separately in both groups of edematous and non edematous patients. In edematous group, both oscillometric and auscultatory methods showed higher systolic BP value compared to arterial method. In auscultatory, it was 1.4mm Hg (+5/-2, P=0.4), while in oscillometric, it was 4.8mm Hg (+8/-1, P=0.02) higher than that in arterial method. In non edematous group, oscillometric and auscultatory showed higher values of systolic BP compared to arterial methods. It was 3 (+5.6/+0.2, P=0.03) in auscultatory and 4.2 (+7/+1, P=0.004) in oscillometric (Table 2, Figure 1).

**Table 2:** Comparison of invasive and non invasive blood pressure measured for both edematous and non edematous groups

	**Devices Mean±SD (CI95%)** ** (differences: Δ ,P value)**	**Edema**	**Non-edema**
Systolic	Arterial/auscultatory	84±17/85±15 (-5/+2) -1.4 mmHg , p= 0.4	86±14/89±15 (-5.6/-0.2) -3 mmHg, p=0.03
	Arterial/oscillometric	84±17/89±15 (-8/+1) -4.8 mmHg, p= 0.02	86±14/91±15(-7/-1) -4.2 mmHg, p=0.004
Diastolic	Arterial/oscillometric	56±8/47±9 (+1.8/+16.5) 9 mmHg, p= 0.03	57±10/55±12 (-0.7/+5) 2.6 mmHg, p=0.2
	Arterial/auscultatory	56±8/54±11 (-6/+10) 1.4 mmHg, p= 0.3	57±10/59±13 (-5/-1) -1.9 mmHg, p= 0.2

Measured diastolic BP by both oscillometric or auscultatory devices in edematous group were lower than that in arterial method, this difference was 9mm Hg(+1.8/16.5, P=0.02) in oscillometric and 2mm Hg(-6/+10, P=0.3) in auscultatory method. Lastly in non edematous group, differences of diastolic BP measured by ausculatatory or oscillometric devices in arterial method were not significant (Table 2, Figure 1).

## Discussion

BP value is considered as a vital sign in both healthy and critical patients, a procedure that can be performed regularly by clinician at home, clinic or intensive care units. Although many studies show that BP measurement is a sensitive technique and its accuracy depends on many factors like resting, taking food before BP measurement or the size of bladder width and length^8^, the type of devices including manual devices or those in oscillometric methods can also affect the accuracy of BP results in children.^9-14^ In addition all oscillometric devices in the market may not have the same accuracy or standard.^15^

Beyond abovementioned factors, biological and vital signs of patients are other variables that can affect the BP measured by oscillometric method.^16^ The age has been mentioned as an effective factor, because systolic and diastolic BPs yielded in mercury manometer were higher than those in oscillometric technique. These discrepancies (mean±SD) were 1.95± 5 and 1.3±4 mmHg for systolic and diastolic BP. The differences were higher for those who aged over 65.^17^ On the other hand, this trend can be reversed in infants and children compared to older people because it was shown that mean± SD of systolic BP was higher (1.7±6) in oscillometric method compared to auscultatory while auscultatory method gave higher diastolic BP, 4±9 mmHg, compared to oscillometric method. In fact oscillometric method overestimates the systolic BP while underestimates the diastolic BP compared to auscultatory method. This may be due to tissue changes like those happened in sclerodermia that affect measurement of BP in both oscillometric and auscultatory method.^6,18^ Obesity is another tissue characteristic that causes overestimation of systolic BP and underestimation of diastolic BP measured by oscillometric in comparison to arterial method in this group.^19,20^

Edema especially in its peripheral form is a common sign that could be met in clinic every day. Edema is the clinical manifestation of an accumulation of fluid within the interstitial spaces of the body.^21^

In this study we tried to find the effect of edema on accuracy of BP obtained by auscultatory, oscillometric and to compare these with arterial values as golden standard of measurement of BP in children candidate for surgery that may be affected by this common tissue property or considerable edematous that configure body shape.

In our study, BP was not different between two groups of children with and without edema measured in all methods.

The most prominent effect of edema on BP happened in diastolic component when measured by oscillometric which gave underestimated results compared to non edematous group that their BP was measured by oscillometric method.

BP values of oscillometric and ausculatatory compared with arterial showed underestimation of diastolic BP values in oscillometric compared to arterial method beyond acceptable standards and overestimation in systolic BP measured by oscillometric compared to arterial method which was not significant. These findings cab be extended to auscultatory method but with lesser severity in diastolic and more prominence in systolic BP. In fact diastolic BP measured by oscillometric in edematous patients was not as much reliable as that measured in children without edema. In fact differences as high as 5 ± 2 mmHg is acceptable in adult when oscillometric method is compared to auscultatory devices as a reference.^1^

Oscillometric method is getting more better and many of studies have compared this method with auscultatory method in healthy and adult group to get more acceptable standards, which should differ less than 5 mmHg.^22,23^

Although our study compared the oscillometric with arterial as invasive standard in children and especially in edematous condition, we guess that this claim can be extended to edematous adult group to confirm the claim that introduces oscillometric method as popular and premature method.^24^

In spite of standards of oscillometric devices usually defined by comparison of auscultatory methods, our study suggests that if this comparison be done by arterial methods, many of these standards can be changeable. Auscultatory method can give a reliable result akin to that of arterial method but auscultatory method depends on many factors such as personnel, device, quiet place and cooperation of patients that all may be inaccessible at once especially in non-cooperating children group. Our study showed that oscillometric devices standards should be improved in edematous children and this feasible and reliable device may show unreliable result in especial conditions. In fact underestimation of diastolic BP as much as 9.2 mmHg in average in edematous is beyond acceptable standards for oscillometric devices.

## Conclusion

Although the oscillometric method is recognized as a feasible and reliable method, clinicians should be aware that in specific conditions like peripheral edema, this method may be non-responsive and underestimate diastolic BP as 9 mmHg in average. This error is beyond the acceptable value for these devices especially in children and infants who have lower BP compared to adults. These errors can affect our clinical decisions against them. In other areas of world, oscillometric is an acceptable method for detecting BP in younger ages.

## Acknowledgements

We would like to thank Ms. Maryam Akhondi for collecting data.

## Ethical issues

The study protocol was approved by the ethics committee of Tabriz University of Medical Sciences.

## Competing interests

Authors declare no conflict of interest in this study.

## References

[1] Smulyan H, Michel E (2011). Safar Blood Pressure Measurement: Retrospective and Prospective Views. AJH.

[2] Perloff D, Grim C, Flack J, Frohlich ED, Hill M, McDonald M (1993). Human blood pressure determination by sphygmomanometry. Circulation.

[3] van Montfrans GA (2001). Oscillometric blood pressure measurement: progress and problems. Blood Press Monit.

[4] American National Standard for Non-Invasive Sphygmomanometers–Part 2: Clinical Validation of Automated Measurement Type ANSI/AAMI/ISO-2:2009.

[5] Bur A, Hirschl MM, Herkner Herkner, Oschatz E, Kofler J, Woisetschläger C, Laggner AN (2000). Accuracy of oscillometric blood pressure measurement according to the relation between cuff size and upper-arm circumference in critically ill patients. Crit care.

[6] Frech TM, Penrod J, Battistone MJ, Sawitzke AD, 
Stults
 
BM
 (2012). Stults BM The Prevalence and Clinical Correlates of an Auscultatory Gap in Systemic Sclerosis Patients. International Journal of Rheumatology.

[7] Nafiu OO, Voepel-Lewis T, Morris M, Chimbira WT, Malviya S, Reynolds Reynolds, PI Kevin (2009). How do pediatric anesthesiologists define intraoperative hypotension?. Pediatric Anesthesia.

[8] (1987). Report of the Second Task Force on Blood Pressure
Control in Children – 1987. Task Force on Blood Pressure Control in Children National Heart, Lung, and Blood Institute, Bethesda, Maryland. Pediatrics.

[9] Park MK, Menard SW, Yuan C (2001). Comparison of auscultatory and oscillometric blood pressures. Arch Pediatr Adolesc Med.

[10] de Man SA, Andre JL, Bachmann H, Grobbee DE, Ibsen KK, Laaser U (1991). Blood pressure in childhood: pooled findings of six European studies. J Hypertens.

[11] Blake KV, Gurrin LC, Evans SF, Newnham JP, Landau LI, Stanley FJ (2000). Reference ranges for blood pressure in preschool Australians, obtained by oscillometry. J Paediatr Child Health.

[12] Ashrafi MR, Abdollahi M, Ahranjani BM, Shabanian R (2005). Blood pressure distribution among healthy schoolchildren aged 6–13 years in Tehran. East Mediterr Health J.

[13] Kelishadi R, Ardalan G, Gheiratmand R, Majdzadeh R, Delavari A, Heshmat R, et  al (2006). Blood pressure and its influencing factors in a national representative sample of Iranian children and adolescents: the CASPIAN Study. Eur J Cardiovasc Prev Rehabil.

[14] Túri   S, Baráth  Á, Boda   K, Tichy   M, Károly   E (2008). Blood pressure reference tables for Hungarian adolescents aged 11–16 years. Kidney Blood Press Res.

[15] O’Brien E, Atkins N (2007). State-of-the-market from the dableducationalorg website. Blood Press Monit.

[16] van Montfrans, Gert A (2001). Oscillometric blood pressure measurement: progress and problems. Blood Pressure Monitoring.

[17] Landgraf J, Wishner SH, Kloner RA (2010). Comparison of automated oscillometric versus auscultatory blood pressure measurement. Am J Cardiol.

[18] Steele Steele (1938). Oscillometric studies of the arteries in scleroderma: Mészáros, K: Acta med Scandinav 95: 522, 1938. Am Heart J.

[19] Umana E, Ahmed W, Fraley MA, Alpert MA (2006). Comparison of oscillometric and intraarterial systolic and diastolic blood pressures in lean, overweight, and obese patients. Angiology.

[20] Lan H, Al-Jumaily AM, Lowe L, Hing W (2011). Effect of tissue mechanical properties on cuff-based blood pressure measurements. Medical Engineering & Physics.

[21] Emmett M, Seldin DW. The Pathophysiology of Edema Formation: General Concepts. In: Seldin D, Giebisch G, editors. Diuretic Agents. Elsevier; 1997. p. 345-358.

[22] Cuckson AC, Reinders A, Shabeeh H, Shennan AH (2002). Validation of the Microlife BP 3BTO-A oscillometric blood pressure monitoring device according to a modified British Hypertension Society protocol. Blood Press Monit.

[23] Coleman A, Freeman P, Steel S, Shennan A (2006). Validation of the Omron 705IT (HEM-759-E) oscillometric blood pressure monitoring device according to the British Hypertension Society protocol. Blood Press Monit.

[24] van Montfrans GA (2001). Oscillometric blood pressure measurement: progress and problems. Blood Press Monit.

